# Lactobacilli decrease the susceptibility of *Salmonella* Typhimurium to azithromycin

**DOI:** 10.1128/spectrum.03497-23

**Published:** 2024-06-25

**Authors:** Lya Blais, Laurence Couture, Isabelle Laforest-Lapointe, Jean-Philippe Côté

**Affiliations:** 1 Département de biologie, Université de Sherbrooke, Sherbrooke, Québec, Canada; University of Manitoba, Winnipeg, Canada

**Keywords:** lactobacilli, *Salmonella *Typhimurium, antibiotic tolerance, azithromycin, pentamidine, genetic screening

## Abstract

Bacteria are involved in numerous interactions during infection and among host-associated microbial populations. *Salmonella enterica* serovar Typhimurium is a foodborne pathogen of great importance as well as a model organism to study interactions within a microbial community. In this study, we found that *S*. Typhimurium becomes tolerant to azithromycin when co-cultured with lactobacilli strains. Similarly, acidified media, from cell-free supernatant of lactobacilli cultures for instance, also induced the tolerance of *S*. Typhimurium to azithromycin. The addition of membrane disruptors restored the normal sensitivity to azithromycin in acidified media, but not when lactobacilli were present. These results suggested that the acidification of the media led to modification in envelope homeostasis, but that a different mechanism promoted the tolerance to azithromycin in the presence of lactobacilli strains. To further understand how lactobacilli strains modify the sensitivity of *S*. Typhimurium to azithromycin, a high-throughput assay was performed using the single-gene deletion collection of the *S*. Typhimurium (1) in co-culture with *Lacticaseibacillus rhamnosus* and (2) in sterile acidic conditions (pH 5.5 media only). As expected, both screens identified genes involved in envelope homeostasis and membrane permeability. Our results also suggest that changes in the metabolism of *S*. Typhimurium induce the tolerance observed in the presence of *L. rhamnosus*. Our results thus highlight two different mechanisms by which lactobacilli induce the tolerance of *S*. Typhimurium to azithromycin.

**IMPORTANCE:**

This study provides valuable insights into the intricate interactions between bacteria during infections and within host-associated microbial communities. Specifically, it sheds light on the significant role of lactobacilli in inducing antibiotic tolerance in *Salmonella enterica* serovar Typhimurium, a critical foodborne pathogen and model organism for microbial community studies. The findings not only uncover the mechanisms underlying this antibiotic tolerance but also reveal two distinct pathways through which strains of lactobacilli might influence *Salmonella*’s response to antibiotics. Understanding these mechanisms has the potential to enhance our knowledge of bacterial infections and may have implications for the development of strategies to combat antibiotic resistance in pathogens, such as *Salmonella*. Furthermore, our results underscore the necessity to explore beyond the direct antimicrobial effects of antibiotics, emphasizing the broader microbial community context.

## OBSERVATION

Bacterial pathogens are often found within polymicrobial communities and thus must interact with other microbes present in the environment. For instance, members of host-associated communities (i.e., microbiota) compete with pathogens and promote resistance to colonization by foreign microbes (1). Not surprisingly, pathogens have adapted to this polymicrobial environment and, for instance, overcome colonization resistance through the use of unique nutrients to persist in their ecological niche (2). There are many examples of the intricate relationships between pathogens and host-associated communities, but we are only beginning to understand the consequences of these interactions. A notorious example of interbacterial relationship is between *Pseudomonas aeruginosa* and *Staphylococcus aureus*, where *P. aeruginosa* profoundly alters the physiology of *S. aureus* and, accordingly, its susceptibility to antimicrobial agents (3–5). Therefore, investigating microbe−microbe interactions could provide new details about bacterial cells found in polymicrobial contexts. Herein, we probed the physiological state of the model intestinal pathogen *Salmonella enterica* serovar Typhimurium by determining its susceptibility against various antibiotics in the presence of lactobacilli and found that *S*. Typhimurium becomes tolerant to azithromycin in the presence of lactobacilli isolates.

**Fig 1:**
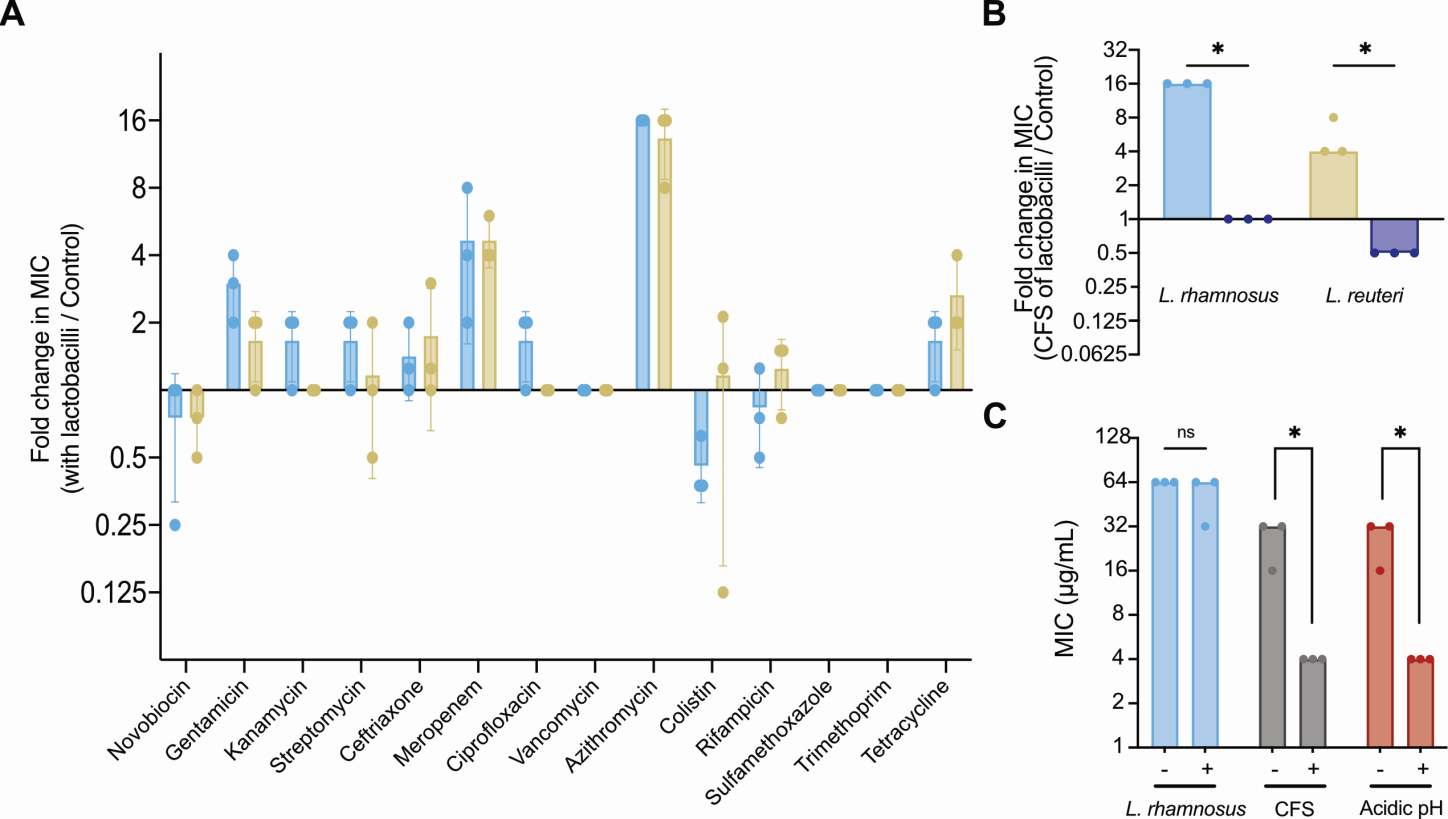
*Salmonella* Typhimurium becomes tolerant to azithromycin when co-cultured with lactobacilli strains. (**A**) Fold change in the MIC of several antibiotics against *S*. Typhimurium in the presence of lactobacilli. Fold change was determined by producing a ratio of the MIC of *S*. Typhimurium in the presence of *L. rhamnosus* (blue) or *L. reuteri* (yellow) and the MIC of *S*. Typhimurium grown by itself against a selected panel of antibiotics *(n* = 2). (**B**) Fold change in the MIC of azithromycin against *S*. Typhimurium in the presence of cell-free supernatant (CFS) (blue/yellow) or neutralized CFS (purple) of lactobacilli compared with the MIC of azithromycin against *S*. Typhimurium in monoculture. The initial pHs were 5.66 and 5.34 for for *L.  rhamnosus* and *L. reuteri* CFS, respectively. CFS was neutralized for both lactobacilli to a pH of 7.00. Wilcoxon–Mann–Whitney U test, * indicates *P* ≤ 0.05 (*n* = 3). (**C**) Impact of the membrane-active compound pentamidine on the MIC of azithromycin for *S*. Typhimurium in the presence of *L. rhamnosus*, CFS, or acidified media. Pentamidine was added at a concentration of 64 µg/mL. Wilcoxon–Mann–Whitney U test, * indicates *P* ≤ 0.05 (*n* = 3).

To explore the impact of lactobacilli on *S*. Typhimurium, we assessed its susceptibility against various antibiotics during co-incubation experiments. Changes in antibiotic sensitivity are a powerful tool to assess the physiological state of the cell (6, 7). We used selective media to determine the minimal inhibitory concentration (MIC) of antibiotics, including relevant antibiotics for treating salmonellosis, such as ciprofloxacin, ceftriaxone, and azithromycin (8, 9), against *S*. Typhimurium co-incubated with one of two lactobacilli strains, *Lacticaseibacillus rhamnosus* LMS2-1 or *Limosilactobacillus reuteri* CF48-3A (Fig. S1A). Co-incubation with both lactobacilli isolates resulted in a 16-fold increase in azithromycin MIC against *S*. Typhimurium (Fig. 1A; Fig. S1B and C; Table S1). Azithromycin is a macrolide antibiotic that targets the ribosome and inhibits protein synthesis. Common resistance mechanisms in *Enterobacteriaceae* notably include target modifications, compound inactivation, and efflux pumps (10). Azithromycin is also dependent on the membrane potential to enter bacterial cells, and thus, modification of envelope integrity or permeability is also a prevalent resistance mechanism (10, 11). Interestingly, *S*. Typhimurium seemed tolerant rather than resistant to the antibiotic in the presence of the lactobacilli, as *S*. Typhimurium cells recovered from the co-cultures at ½ MIC (16 µg/mL) reverted to their initial susceptibility to azithromycin when incubated in monocultures (MIC of 4 µg/mL; Table S1). In addition, heat-killed lactobacilli cells did not increase the MIC to azithromycin against *S*. Typhimurium (Table S1).

Cell-free supernatant (CFS) from both lactobacilli strains also induced azithromycin tolerance in *S*. Typhimurium (Fig. 1B). As expected, CFS from the lactobacilli strains was acidified compared with the media used for the experiment (pH of 5.66 for *L. rhamnosus* and 5.34 for *L. reuteri*). When neutralized CFS was used, the MICs returned to normal (Fig. 1B). In addition, replacement of CFS by acidified media (BHI at pH 5.5) in combination with *S*. Typhimurium showed a similar increase in MIC (Table S1), suggesting that the decrease in pH caused the observed tolerance against azithromycin as expected (12). It is important to note that azithromycin is stable in acidic conditions (9), and thus, the tolerance to the antibiotic is not likely due to compound breakdown. These results suggest that uptake of azithromycin is decreased in the presence of the lactobacilli strains because of the acidification of media, as azithromycin is dependent on the membrane potential to enter bacterial cells (11).

To confirm that azithromycin tolerance was due to reduced uptake and because azithromycin can be potentiated by decreasing the permeability of the outer membrane (10, 13, 14), we conducted co-incubation assays in the presence of two outer membrane destabilizing agents: pentamidine and EDTA (14, 15), both of which bind to lipopolysaccharides. The addition of pentamidine (Fig. 1C; Fig. S2) or EDTA (Fig. S3) still resulted in an increased MIC of azithromycin against *S*. Typhimurium when co-cultured with *L. rhamnosus*. However, when CFS of *L. rhamnosus* or acidified media were used, pentamidine and EDTA decreased the MIC of azithromycin to values observed for monocultures of *S*. Typhimurium. These results suggest the existence of two discrete and separate mechanisms contributing to the observed phenotype. As expected, the acidification of the media most likely reduced the uptake of azithromycin, which could be reversed by increasing envelope permeability (12, 13, 16). However, in the presence of lactobacilli, a distinct mechanism emerged as a strong driver of the tolerance of *S*. Typhimurium to azithromycin.

**Fig 2:**
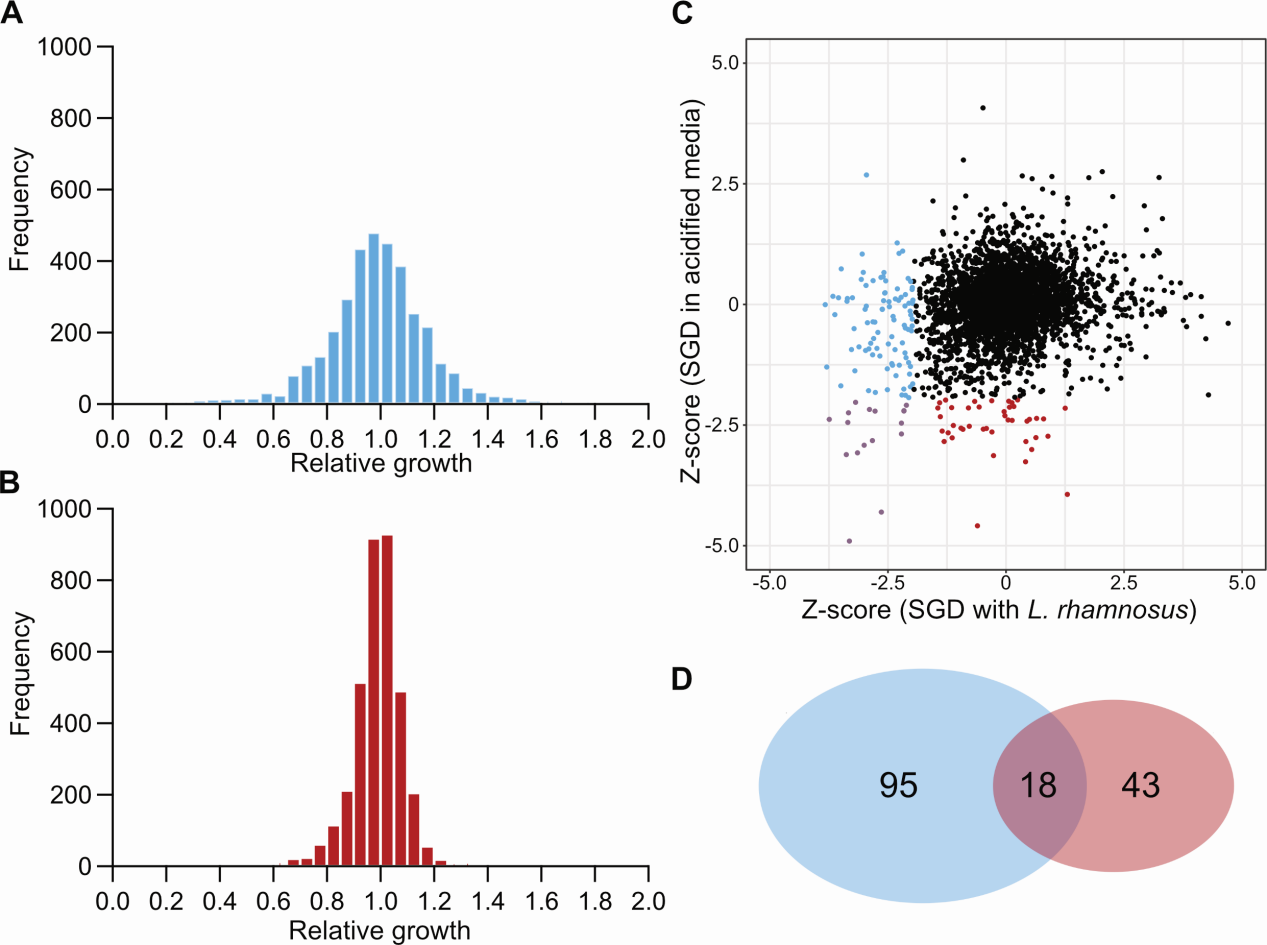
Growth of the single-gene deletion (SGD) library at high azithromycin concentrations in the presence of *L. rhamnosus* or acidified pH. The SGD library was grown in the presence of (**A**) *L. rhamnosus* (blue) or (**B**) acidified media (red) and with 128 µg/mL of azithromycin (16× MIC in tested conditions). The growth of each mutant in the presence of azithromycin was compared with controls without azithromycin to yield a relative growth (a.u.) value, where a value of 1 indicates that azithromycin did not affect the growth of the mutant. (**C**) Relative growth values were converted to Z-scores. We selected hits corresponding to genes that were significantly different from the population (Z-score, <−1.96, *P* ≤ 0.05) in the presence of *L. rhamnosus* (blue), acidified media (red), or in both conditions (purple). (**D**) Venn diagram of selected hits.

To investigate the response of *S*. Typhimurium to the presence of lactobacilli isolates or acidified media, we reproduced the co-culture experiments on a larger scale, using a genome-wide single-gene deletion (SGD) collection of *S*. Typhimurium (17) and either high-density cultures of *L. rhamnosus* or acidified media, both in the presence or absence of azithromycin. In our high-throughput experiments, the MIC of azithromycin against *S*. Typhimurium alone was higher; therefore, we used a concentration of 128 µg/mL of azithromycin in our high-throughput assay to be representative of our initial observation (16-fold increase in MIC). At this concentration, *S*. Typhimurium only grows when *L. rhamnosus* is present or in acidified media, but not in monoculture. Mutants showing a growth defect specifically when treated with high concentrations of azithromycin indicate that their deleted gene is essential for the tolerance phenotype. We compared the growth of the SGD library with and without azithromycin in the presence of either (i) *L. rhamnosus* or (ii) acidified media. The two conditions showed different responses to the presence of azithromycin, with the presence of *L. rhamnosus* having a bigger impact on *S*. Typhimurium (Fig. 2A), corroborating our hypothesis that different mechanisms are involved in the increased tolerance. We transformed screening values into Z-scores and selected gene deletions that were significatively different than the population (*P* ≤ 0.05) (Fig. 2B; Table S3). Although we did not necessarily observe the same hits, both screens were enriched for gene deletions (*ldcA*, *mdoG*, *pal*, *pgm*, *prc, rfaP*, *rfaY*, *wecC*, *wecG*, and *yceG* for instance) that have been shown to disrupt the permeability of the outer membrane in *Escherichia coli* (18). In addition, in the presence of *L. rhamnosus*, the growth of both gene deletions from the two-component systems *envZ/ompR* and *phoP/phoQ* was impaired in the presence of azithromycin. The prevalence in genes affecting membrane homeostasis supports the effect of membrane disruptors in acidified media. In the presence of *L. rhamnosus*, additional gene deletions (*nuo* genes, *sdhAB*, *sucB*, *acnB*, *pfkB*, *rpoN*, *glnK*, and *arcA* for instance) affecting core metabolic processes also impaired the tolerance of *S*. Typhimurium to azithromycin. Our results suggest that, in the presence of lactobacilli, *S*. Typhimurium undergoes changes in its metabolism leading to tolerance to azithromycin that prevails the decreased membrane permeability observed in acidified media.

Further investigations of the interaction between *S*. Typhimurium and lactobacilli strains are necessary to fully understand the molecular basis behind how lactobacilli isolates can induce a change in the susceptibility of the pathogen to azithromycin. In light of the escalating antibiotic resistance worldwide and the considerable obstacles to developing novel antibiotic molecules, our results underscore the urgency of delving deeper into the intricate dynamics between pathogens, such as *S*. Typhimurium, and common human-associated bacteria, such as lactobacilli, as they have the potential to significantly impact the effectiveness of antibiotic therapies.

## SUPPLEMENTAL MATERIAL

Supplemental material spectrum.03497-23-s0001.docx Fig. S1 to S3; Tables S1 and S2.

Table S3 spectrum.03497-23-s0002.xlsx Relative growth and Z-score values for the complete SGD collection.

ASM does not own the copyrights to Supplemental Material that may be linked to, or accessed through, an article. The authors have granted ASM a non-exclusive, world-wide license to publish the Supplemental Material files. Please contact the corresponding author directly for reuse.
