# Supplementary material for: Lactobacilli decrease the susceptibility of Salmonella Typhimurium to azithromycin
Source: Microbiol Spectr. 2024 Jun 25;12(8):e03497-23. doi: 10.1128/spectrum.03497-23 (PMC11302071; doi:10.1128/spectrum.03497-23)
Supplement: Supplemental material — Fig. S1 to S3; Tables S1 and S2. [file spectrum.03497-23-s0001.docx]

**Supplemental Materials**

**Lactobacilli decrease the susceptibility of *Salmonella* Typhimurium to azithromycin**

**Authors:** Lya Blais¹*, Laurence Couture¹*, Isabelle Laforest-Lapointe¹ & Jean-Philippe Côté¹^#^

**Affiliations :**

¹Département de biologie, Université de Sherbrooke, Sherbrooke, Québec, Canada, J1K 2R1

# Correspondence may be addressed to:

Jean-Philippe Côté

Tel :1-819-821-8000 ext. 65280

Email: jp.cote@usherbrooke.ca

**This Supplemental Material includes:**

Materials and methods

Supplementary Figures 1 to 3

Supplementary Tables 1 and 3

References

**Materials and Methods**

Bacterial strains and growth conditions

All experiments in this study were performed with *Salmonella enterica* serovar Typhimurium strain 14028s or the described single-gene deletion library (SGD library) of *S.* Typhimurium 14028s (1). *Limosilactobacillus reuteri* CF48-3A, *Lacticaseibacillus rhamnosus* LMS2-1 and *Staphylococcus aureus* 130 were obtained from the Human Microbiome Project (2) and were cultured in Brain-Heart infusion (BHI) broth prepared according to Lau *et al* (3). All strains were grown at 37 ℃. The SGD library was maintained in BHI broth supplemented with kanamycin at a concentration of 50 mg/ml.

Products and solutions

A selective panel of antibiotic compounds was selected to cover various antibiotic classes. Azithromycin, meropenem and sulfamethoxazole were obtained from Sigma Aldrich and diluted in dimethyl sulfoxide (DMSO). Tetracycline, novobiocin, cefuroxime, ceftriaxone and colistin were also obtained from Sigma Aldrich but were diluted in water. Rifampicin, erythromycin, nalidixic acid and trimethoprim were obtained from Bio Basic and diluted in DMSO. Streptomycin, gentamicin, ampicillin, kanamycin, ciprofloxacin and vancomycin were also obtained from Bio Basic but were diluted in water. Lastly, apramycin was obtained from Alfa Aesar and was diluted in water. Ethylenediamine tetraacetic acid (EDTA) was obtained from Fisher Bioreagents and diluted in water. Pentamidine isethionate salt (Sigma Aldrich) was diluted in DMSO. All antibiotics and pentamidine were solubilized initially at 25 mg/mL.

Minimal inhibitory concentration assay

Cells were grown overnight in BHI, and diluted 1:1000 in fresh BHI. Cells were then added in 96-well plates containing twofold dilution series of the antibiotics starting at 256 µg/mL. After incubating for 18h at 37˚C, optical density at 600nm was measured on a Spark 20M multimode microplate reader (TECAN). When indicated, EDTA or pentamidine was supplemented at a final concentration of 2 mM and 64 µg/mL, respectively. The MIC determination assay was also performed in acidic conditions as previously stated. Acidified BHI (pH of 5.5) was prepared with a solution of HCl 1N and used to dilute the different antibiotics to be tested in serial dilutions in 96-well plate.

Co-culture minimal inhibitory concentration assay

The MIC of *S.* Typhimurium against various antibiotics was determined in the presence of *L. reuteri* CF48-3A or *L. rhamnosus* LMS2-1. Overnight cultures of lactobacilli strains were grown in BHI for 18h at 37℃ with agitation, reaching OD_600_ values of ~ 1.0-1.2 (~10^8^ CFU/ml, determined by bacterial counts on MRS agar). *S*. Typhimurium was also grown overnight, reaching OD_600_ value of ~1.5 (~10^9^ CFU/mL), then diluted 1:1000 in fresh BHI. The co-culture experiments were performed by mixing the diluted culture of *Salmonella* with overnight cultures of the lactobacilli strains in a 1:1 volume ratio into 96-well plates containing twofold dilutions of the antibiotics. The co-culture plates were then incubated for 18h at 37℃. After incubation, final pH of the co-cultures and 5 µL of each well were plated on *Salmonella Shigella* (SS) agar, which is selective for Gram-negative bacteria. The agar plates were incubated overnight at 37 ℃, after which the MIC of *S.*Typhimurium was determined visually, as the lowest concentration inhibiting visible growth. The method is shown in Supplementary Figure S1A. When needed, EDTA or pentamidine were also added to the wells as described in the MIC assays.

Final bacterial counts of the co-cultures were also determined by serial dilution by plating on MRS agar and SS agar. For all azithromycin concentrations, final lactobacilli counts were 2x10^8^ CFU/mL for *L. rhamnosus* and 4x10^8^ CFU/mL for *L. reuteri* on average. *S.*Typhimurium typically reached from ~5x10^9^ CFU/mL (at ½ MIC) to ~10^11^ CFU/mL in wells below the MIC. In wells above the MIC, we typically recovered ~10^7^ CFU/mL.

For co-cultures with the cell free supernatant (CFS) of lactobacilli strains, lactobacilli cultures were grown for 18h at 37 ℃ with agitation. The overnight cultures were then centrifugated at 4,000 rpm for 10 min. The resulting supernatant was collected and filtered with a sterile 0.22 µm syringe filter. The sterile supernatants were then added to the 96-well plates containing the diluted *S*. Typhimurium and the antibiotics, as described above. For co-cultures with heat-killed strains, cultures were grown for 18h at 37 °C with agitation. After the incubation, strains were subjected to a heat treatment of 20 min at 70 °C, followed by two rounds of 5 min centrifugation at 13,000 rpm and washing in 1X PBS. Washed pellet was resuspended in BHI before being added to the 96-well plates containing the diluted *S.*Typhimurium and the antibiotics, as described above.

Assessment of antibiotic tolerance/resistance

The MIC of azithromycin against *S*. Typhimurium after a co-culture with *L. rhamnosus* was determined as described in the previous section. Following the incubation on *Salmonella Shigella* (SS) agar, *S*. Typhimurium cells that grew at ½ MIC of azithromycin (typically 16 or 32 µg/mL) were harvested using an inoculating loop and grown for 18h at 37°C with agitation in fresh BHI. Recovered *S*. Typhimurium were then diluted 1:1000 in BHI and added into a 96-well plate containing increasing concentrations of azithromycin. The plate was incubated overnight at 37°C, after which 5 µl of each well were plated on SS agar. The agar plate was incubated overnight at 37°C and the MIC of recovered *S*. Typhimurium was determined. Following this, recovered *S.* Typhimurium growing at high concentrations of azithromycin (16 or 32 µg/mL) are defined as resistant cells, while recovered *S.* Typhimurium growing only at lower concentrations (2 or 4 µg/mL) are defined as tolerant cells.

Checkerboard broth microdilution assay

The MIC of *S.* Typhimurium against azithromycin was determined in presence of increasing concentrations of pentamidine and a co-culture with either *L. rhamnosus*, CFS of *L. rhamnosus* or acidified media (BHI pH 5.5). For this, strains were grown for 18h at 37 °C with agitation. *S.* Typhimurium was diluted 1:1000 in BHI and mixed with the overnight culture of *L. rhamnosus*, CFS of *L. rhamnosus* (which was obtained as described in the previous section) or acidified media in 96-well plates in a 1:1 ratio. Azithromycin and pentamidine were serially diluted as described for the MIC assays and were added at concentrations ranging from 0 to 256 µg/ml. The co-culture plates were then incubated for 18h at 37 ℃. After the incubation, 5 µL of each well was plated on *Salmonella Shigella* (SS) agar, which is selective for Gram-negative bacteria. The agar plates were incubated overnight at 37 ℃, after which the MIC of *S.* Typhimurium in presence of various concentrations of pentamidine was determined visually.

High-throughput co-culture assay using the SGD collection

*S*. Typhimurium SGD collection was grown for 18h at 37 ℃ in 384-well plates filled with 50 µL of BHI. *L. rhamnosus* LMS2-1 was grown separately in 25 ml of BHI at 37 ℃ for 18h. The co-culture plates were then prepared by adding 25 µL of overnight *L. rhamnosus* culture and 25 µL of BHI supplemented with 256 µg/mL of azithromycin in 384-well plates (final concentration of 128 µg/mL in 50 µl). The SGD collection was finally inoculated to the 384-well co-culture plates with the Rotor HDA (Singer Instruments, UK). This setup transfers ~0.5 µl of the SGD culture into the new 384-well plates. The co-culture plates were incubated for 18h at 37 ℃. The viability of *S*. Typhimurium was then determined by pinning from the 384-well co-culture plates onto SS agar, which were incubated overnight at 37 ℃. Images of each plate were taken with the Phenobooth (Singer Instruments, UK) for analysis. This high-throughput method was also performed under acidic pH conditions. In this case, the 25 µL of the overnight culture of *L. rhamnosus* was replaced by 25 µL of BHI pH 5.5.

Images of the bacterial growth on SS agar plates were analyzed by an in-house analysis pipeline, as described previously (4). Briefly, imaged plates were divided into 384 region-of-interests (roi) to isolate each colony and the density within each roi was measured using Fiji (5). Density values were then normalized with the *lowess* ­in R (24). We determined to relative growth of every mutant of the collection for a given treatment (the presence of *L. rhamnosus* or acidified media) by calculation the ratio of the normalized density value with azithromycin and without azithromycin. A relative growth around 1 signifies that the gene deletion growth is similar with or without azithromycin, while a relative growth < 1 means that the growth of gene deletion is impaired in the presence of azithromycin. Relative growth values were then converted to Z-scores, Z score = (relative growth– screen mean)/screen sd. Based on the statistical distribution obtained from the Z-scores, we selected the gene deletions that were the most distant from the dataset average (p-values ≤0.05).

**Figures & Tables**

**
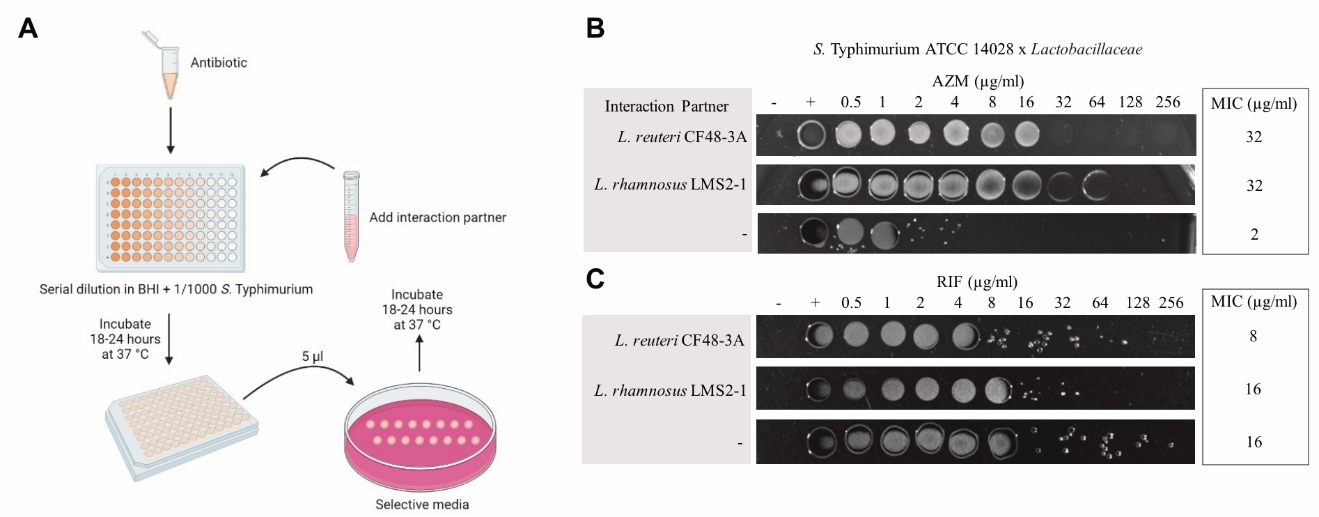
**

**Figure S1. Co-culture minimal inhibitory concentration assay.** (A) Summary of the method used to determine the susceptibility of *S*. Typhimurium to different antibiotics following a co-culture. Co-cultures were performed in BHI media. After incubation, *S.* Typhimurium is selected by spotting 5 µL of the co-cultures on SS agar. (B) Changes in the susceptibility of *S*. Typhimurium to azithromycin when co-cultured with lactobacilli. MIC against *S*. Typhimurium is shown on SS agar either alone with the antibiotic or in combination with lactobacilli. Final pH decreases from 8.0 to 5.6 in the co-culture with *L. rhamnosus* and from 7.7 to 5.8 with *L. reuteri* as azithromycin concentration increases from 0 to 256 µg/ml. (C) Strain combinations performed with rifampicin which is not impacted by the co-culture.

**
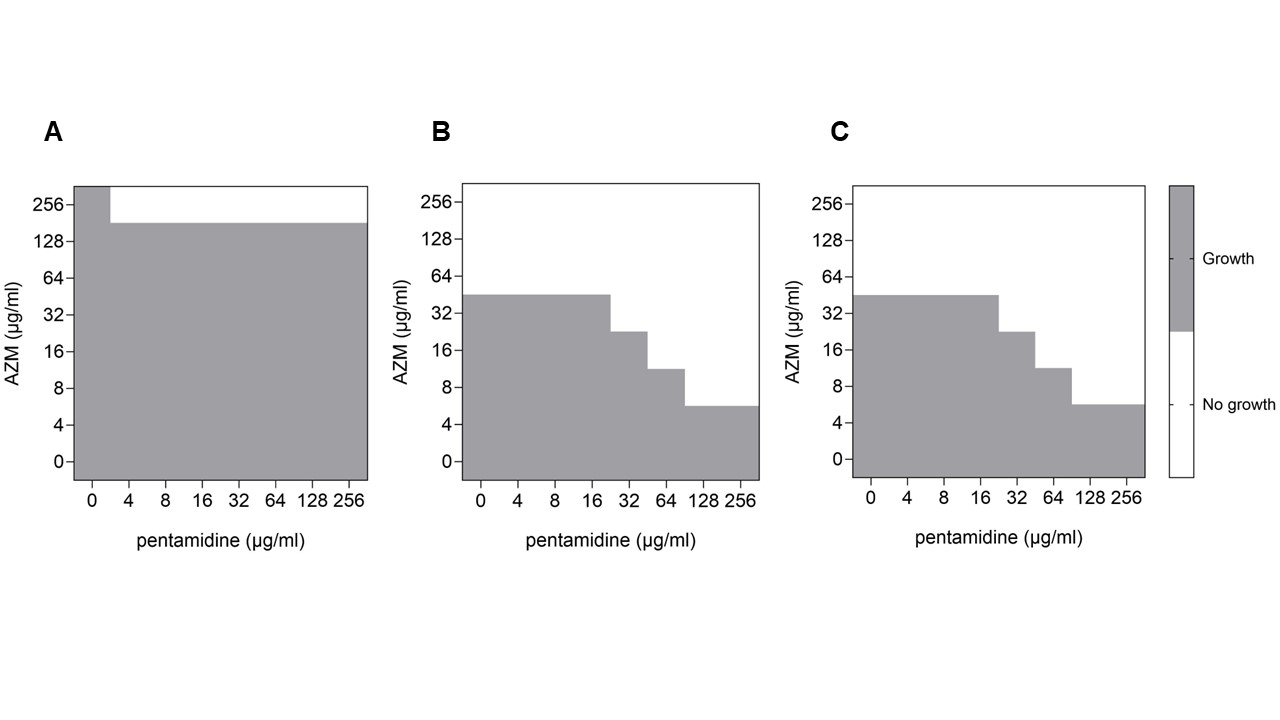
**

**Figure S2. Impact of the pentamidine concentration on the MIC.** Impact of increasing concentrations of pentamidine on the MIC of azithromycin against *S.* Typhimurium co-cultured with *L. rhamnosus* (A), with *L. rhamnosus* derived CFS (B), or with acidified media (C). Grey shows growth of *S.* Typhimurium on the SS plates after co-culture experiments while white means no growth.

**
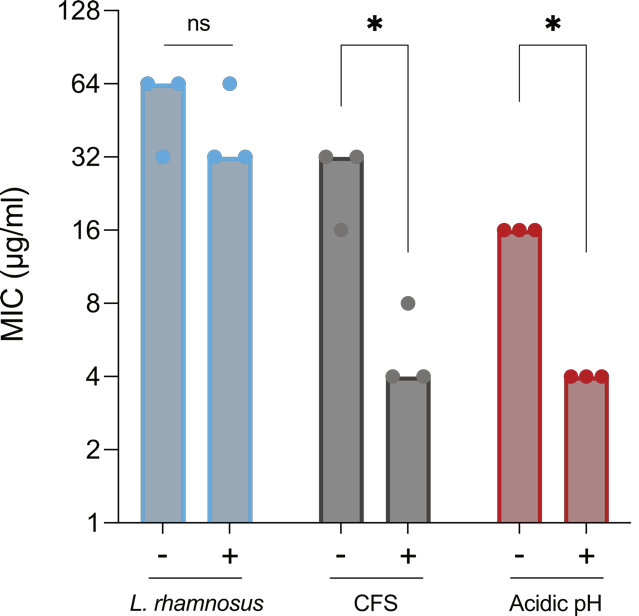
**

**Figure S3. Addition of EDTA in co-culture MIC assays.** Impact of EDTA on the MIC of azithromycin against *S.* Typhimurium in presence of *L. rhamnosus* culture, *L. rhamnosus* derived CFS or an acidified media. EDTA was added at a concentration of 2 mM. Wilcoxon-Mann-Whitney U test, * indicates a p-value ≤ 0.05.

**Table S1. MIC of azithromycin against *S.* Typhimurium in various conditions.**

| **Strain** | **Combination** | **MIC of azithromycin (µg/mL)** |
| --- | --- | --- |
| *S.* Typhimurium | BHI (pH 7.4) | 2-4 |
|  | *L. rhamnosus* | 32-64 |
|  | *L. rhamnosus* CFS | 32-64 |
|  | *L. rhamnosus* neutralized CFS | 4-8 |
|  | *L. reuteri* | 32-64 |
|  | *L. reuteri CFS* | 32-64 |
|  | *L. reuteri* neutralized CFS | 2-4 |
|  | BHI (pH 5.5) | 32-64 |
|  | Heat-killed *L. rhamnosus* | 2 |
| Recovered *S*. Typhimurium* | BHI (pH 7.4) | 4 |

* *Salmonella* cells that survived ½ MIC treatments in the presence of *L. rhamosus* or *L. reuteri* were recovered from SS plates and the MIC against recovered bacteria was determined in fresh BHI.

**Table S2. MIC of azithromycin against *S.* Typhimurium in different combination with the addition of membrane-destabilizing agents**

| **Strain** | **Combination** | | **MIC of azithromycin (µg/mL)** | | |  |
| --- | --- | --- | --- | --- | --- | --- |
|  |  |  | | + EDTA | + pentamidine | |
| *S.* Typhimurium | BHI (pH 7.4) | 2 | | 0.5 | 0.5 | |
|  | *L. rhamnosus* | 32-64 | | 32-64 | 32-64 | |
|  | *L. reuteri* | 16-64 | | 32-64 | 32-64 | |
|  | BHI (pH 5.5) | 16-32 | | 4 | 4 | |
|  | *L. rhamnosus* CFS | 16-32 | | 4-8 | 4 | |

Note: EDTA was used at 2 mM and pentamidine at 64 µg/mL.

**Table S3. Relative growth and Z-score values for the complete SGD collection in the presence of *L. rhamnosus* or in acidified conditions.** See Excel file.

**References**

1. Porwollik S, Santiviago CA, Cheng P, Long F, Desai P, Fredlund J, Srikumar S, Silva CA, Chu W, Chen X, Canals R, Reynolds MM, Bogomolnaya L, Shields C, Cui P, Guo J, Zheng Y, Endicott-Yazdani T, Yang H-J, Maple A, Ragoza Y, Blondel CJ, Valenzuela C, Andrews-Polymenis H, McClelland M. 2014. Defined Single-Gene and Multi-Gene Deletion Mutant Collections in *Salmonella enterica* sv Typhimurium. PLoS ONE 9:e99820.

2. Turnbaugh PJ, Ley RE, Hamady M, Fraser-Liggett CM, Knight R, Gordon JI. 2007. The human microbiome project. Nature 449:804–810.

3. Lau JT, Whelan FJ, Herath I, Lee CH, Collins SM, Bercik P, Surette MG. 2016. Capturing the diversity of the human gut microbiota through culture-enriched molecular profiling. Genom Med 8:72.

4. French S, Mangat C, Bharat A, Côté J-P, Mori H, Brown ED. 2016. A robust platform for chemical genomics in bacterial systems. Mol biol cell 27:1015–1025.

5. Schindelin J, Arganda-Carreras I, Frise E, Kaynig V, Longair M, Pietzsch T, Preibisch S, Rueden C, Saalfeld S, Schmid B, Tinevez J-Y, White DJ, Hartenstein V, Eliceiri K, Tomancak P, Cardona A. 2012. Fiji: an open-source platform for biological-image analysis. 7. Nat Methods 9:676–682.
